# The Product Specificities of Maize Terpene Synthases TPS4 and TPS10 Are Determined Both by Active Site Amino Acids and Residues Adjacent to the Active Site

**DOI:** 10.3390/plants9050552

**Published:** 2020-04-26

**Authors:** Tobias G. Köllner, Jörg Degenhardt, Jonathan Gershenzon

**Affiliations:** 1 Max Planck Institute for Chemical Ecology, Hans-Knöll-Strasse 8, D-07745 Jena, Germany; gershenzon@ice.mpg.de; 2 Institute for Pharmacy, Martin-Luther-University Halle-Wittenberg, Hoher Weg 8, D-06120 Halle/Saale, Germany; joerg.degenhardt@pharmazie.uni-halle.de

**Keywords:** sesquiterpene synthase, terpene synthase reaction mechanism, β-farnesene, α-bergamotene, 7-*epi*-sesquithujene, β-bisabolene, site-directed mutagenesis, *Zea mays*

## Abstract

Terpene synthases make up a large family of enzymes that convert prenyl diphosphates into an enormous variety of terpene skeletons. Due to their electrophilic reaction mechanism—which involves the formation of carbocations followed by hydride shifts and skeletal rearrangements—terpene synthases often produce complex mixtures of products. In the present study, we investigate amino acids that determine the product specificities of the maize terpene synthases TPS4 and TPS10. The enzymes showed 57% amino acid similarity and produced different mixtures of sesquiterpenes. Sequence comparisons and structure modeling revealed that out of the 43 amino acids forming the active site cavity, 17 differed between TPS4 and TPS10. While combined mutation of these 17 residues in TPS4 resulted in an enzyme with a product specificity similar to TPS10, the additional mutation of two amino acids next to the active site led to a nearly complete conversion of TPS4 into TPS10. These data demonstrate that the different product specificities of TPS4 and TPS10 are determined not only by amino acids forming the active site cavity, but also by neighboring residues that influence the conformation of active site amino acids.

## 1. Introduction

The enormous number of terpene structures found in nature can be attributed to the enzyme class of terpene synthases (TPS). These catalysts accept the ubiquitous prenyl diphosphates geranyl diphosphate (GPP), (*E,E*)-farnesyl diphosphate (FPP) and (*E,E,E*)-geranylgeranyl diphosphate (GGPP) as substrates and convert them into the basic monoterpene, sesquiterpene and diterpene carbon skeletons, respectively [1]. The product diversity of terpene synthases can be explained by their electrophilic reaction mechanism, which involves the formation of reactive carbocationic intermediates. These carbocations can undergo different cyclizations, hydride shifts and other rearrangements until the reaction is terminated by deprotonation or water addition [1,2,3].

**Figure 1:**
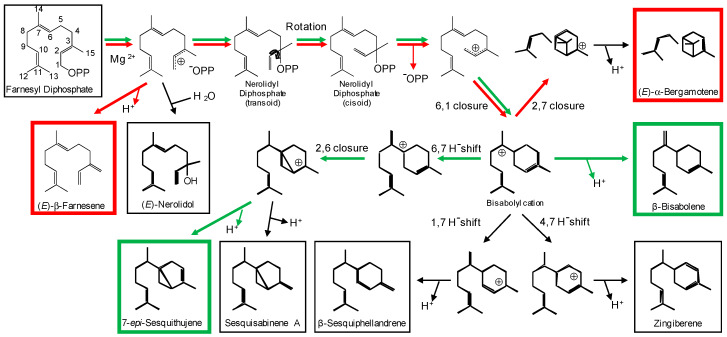
Comparison of the reaction mechanisms catalyzed by TPS4 and TPS10. The major TPS4 products 7-*epi*-sesquithujene and β-bisabolene are marked by green boxes, while the major TPS10 products (*E*)-β-farnesene and (*E*)-α-bergamotene are marked by red boxes. Green and red arrows indicate main steps in the reaction sequences of TPS4 and TPS10, respectively.

We previously characterized two sesquiterpene synthases TPS4 and TPS10 that are involved in volatile terpene formation in maize (*Zea mays*) [4,5,6]. While TPS4 is mainly expressed in maize husk leaves and forms the major constitutive volatiles emitted from this tissue, TPS10 contributes to the herbivory-induced sesquiterpene production in maize seedlings. TPS4 has been reported to produce a mixture of more than 22 sesquiterpenes, with 7-*epi*-sesquithujene and β-bisabolene being the major compounds [4]. The reaction catalyzed by TPS4 starts with the Mg^2+^-dependent ionization of the substrate (*E,E*)-FPP (Figure 1). The resulting (*E,E*)-farnesyl cation is then isomerized to the (*Z,E*)-isomer, presumably via the formation of nerolidyl diphosphate as an intermediate. A subsequent C6-C1 ring closure (numbering as for FPP) leads to the bisabolyl cation, which can be deprotonated to form the neutral product β-bisabolene. Alternatively, the bisabolyl cation can undergo a C6-C7 hydride shift and a C2-C6 ring closure. Deprotonation of the resulting sesquithujyl cation leads to the second major product 7-*epi*-sesquithujene [4]. TPS10, with 57% amino acid similarity to TPS4, produces seven sesquiterpenes that are also formed as minor products by TPS4 [7]. However, the major TPS10 products, (*E*)-α-bergamotene and (*E*)-β-farnesene, are derived by a C2-C7 ring closure of the bisabolyl cation and subsequent deprotonation, or by deprotonation of the initially produced farnesyl cation, respectively [7]. In general, multiple product formation by terpene synthases is assumed to be the result of variable starting conformations of the substrate and alternative conformational changes of reaction intermediates guided by the active site contour [8,9,10]. For example, structure modeling, in silico substrate docking, and site-directed mutagenesis of active site residues suggested that individual intermediates of the reaction sequence of TPS4 can be bound with alternative conformations in the active site cavity [10].

Although many terpene synthases have been characterized during the last three decades and several studies have addressed the role of single amino acids in TPS catalysis, a precise prediction of product specificities based on TPS amino acid sequences is not yet possible [1]. Thus, the analysis of structure-function relationships in TPS enzymes is an important research goal that helps to understand the evolution of terpene synthases and may allow the rational design of TPS enzymes with novel functions in the future. In the present study, we used structure modeling and sequence comparisons to identify amino acids that determine the different product specificities of maize TPS4 and TPS10. Active site residues and neighboring amino acids were investigated using site-directed mutagenesis. The resulting mutant proteins were assayed in vitro, and product profiles were analyzed by gas chromatography-mass spectrometry. The data showed that active site amino acids in combination with a few adjacent residues determine the product specificities of TPS4 and TPS10.

## 2. Results

### 2.1. The Active Sites of TPS4 and TPS10 Differ in 17 Amino Acid Residues

**Figure 2:**
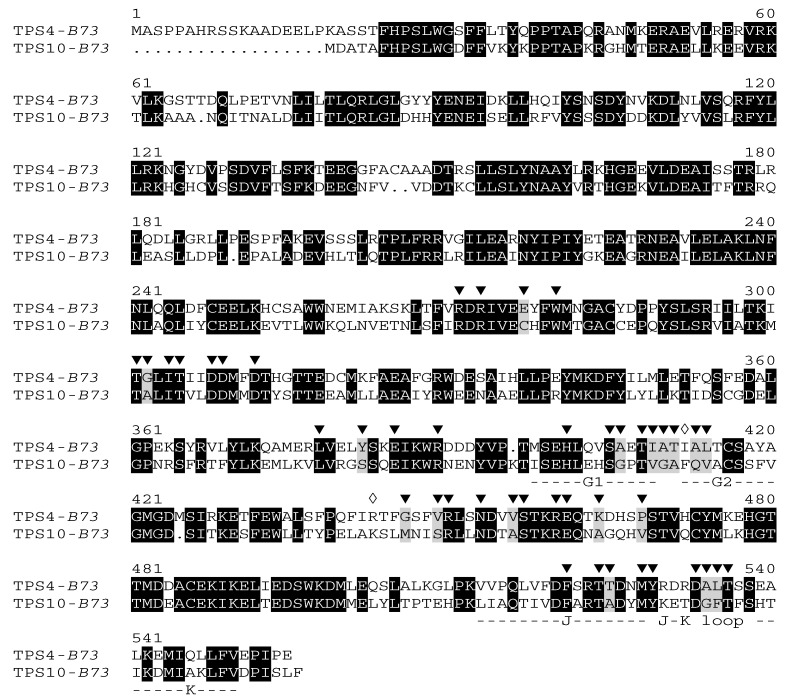
Comparison of the deduced amino acid sequences of TPS4 and TPS10. Amino acids identical in both proteins are marked by black boxes. Amino acids situated at the surface of the active site cavity are highlighted by arrowheads. The white diamonds indicate the two mutated residues outside the active site cavity. Helices J and K and the J-K loop are indicated.

Based on prior modeling, the active site cavity of TPS10 was found to be lined by 43 amino acids located in the C-terminal domain of the enzyme [7]. To compare TPS10 to TPS4, a homology-based structure model of TPS4 was generated using the three-dimensional structure of 5-*epi*-aristolochene synthase from *Nicotiana tabacum* [11] as template. The overall structures of the C-terminal domains of both proteins were found to be highly conserved. Moreover, the model showed that the active site of TPS4 is formed by residues corresponding to the same 43 amino acids identified in TPS10 (Supplemental Figure S1), although 17 out of the 43 residues differed between the two enzymes (Figure 2).

### 2.2. Combined Mutation of the 17 Active Site Residues in TPS4 Alters the Product Specificity of the Enzyme Towards That of TPS10

**Figure 3:**
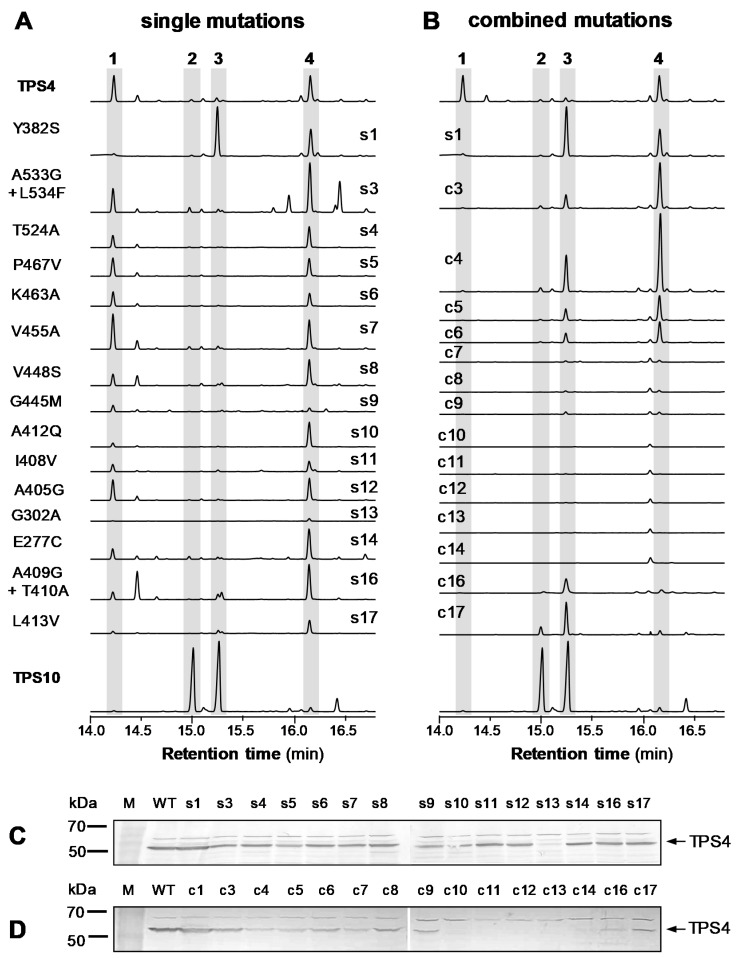
Site-directed mutagenesis of 17 active site amino acids differing between TPS4 and TPS10. The effects of single mutations, s1–s17 (**A**) and combined mutations, c1–c14 (**B**), on product specificity were studied. Genes were heterologously expressed in *Escherichia coli* and partially purified enzymes were incubated with (*E,E*)-farnesyl diphosphate (FPP) in the presence of the cofactor Mg^2+^. Total ion chromatograms of gas chromatography-mass spectrometry (GC-MS) analyses of the terpene products are shown. The sesquiterpene olefins were identified by mass spectrometry and comparison of mass spectra and retention times to those of authentic standards. 1, 7-*epi*-sesquithujene; 2, (*E*)-α-bergamotene; 3, (*E*)-β-farnesene; 4, β-bisabolene. The expression of soluble terpene synthase (TPS) enzymes was tested by western blot analysis of *E. coli* raw protein extracts using a TPS4-specific antibody. Data for single mutants (**C**) and combinatorial mutants (**D**) are shown.

In order to study the roles of the 17 differing active site amino acids in defining the respective product specificities of TPS4 and TPS10, we used TPS4 as template and created a number of mutants containing either a single amino acid switch to that of TPS10 or, in case of neighboring residues, a pair of mutated amino acids. The resulting 15 mutant proteins (Supplemental Table S1) were heterologously produced in *Escherichia coli* and assayed with (*E,E*)-FPP as substrate. Although many of the mutants created showed altered product profiles and one (TPS4 Y382S) produced mainly (*E*)-β-farnesene, none of them had a product specificity comparable to that of TPS10 with (*E*)-α-bergamotene and (*E*)-β-farnesene as major products (Figure 3A). Next, a combined mutant TPS4-c17 that possessed all 17 amino acid changes (Supplemental Table S1) was constructed stepwise by introducing a single mutation at a time into TPS4 and using each mutant gene produced as a template for subsequent mutagenesis PCR. The mutant TPS4-c17 and the 14 combined mutants made in between TPS4 and TPS4-c17 (Supplemental Table S1) were all heterologously expressed and tested as described above. In contrast to the single mutants, which all showed some TPS activity with one exception, four of the combined mutants (TPS4-c7, TPS4-c8, TPS4-c9, TPS4-c14) were nearly or completely inactive, although recombinant proteins could be detected with TPS4-specific antibodies (Figure 3B,D). Four other mutants (TPS4-c10, TPS4-c11, TPS4-c12, TPS4-c13) could not be expressed as soluble proteins in *E. coli* (Figure 3D), indicating that the introduced combinations of amino acid changes likely influenced the overall folding and stability of the proteins. However, the combination of all 17 amino acid changes resulted in the mutant protein TPS4-c17 that produced (*E*)-β-farnesene as the main product and substantial amounts of (*E*)-α-bergamotene as a minor product (Figure 3B; Table 1). Kinetic characterization of the mutant TPS4-c17 and the wild-type enzymes TPS4 and TPS10 revealed comparable *K*_m_ values for the substrate (*E,E*)-FPP (Table 2). The *k*_cat_ value of TPS4-c17, however, was only approximately 43% and 2% of the *k*_cat_ values of TPS4 and TPS10, respectively (Table 2; Supplemental Figure S2).

### 2.3. The Differences in Product Specificity of TPS4 and TPS4-c17 Are not Determined Exclusively by Amino Acids in the Helices G1 and G2

**Figure 4:**
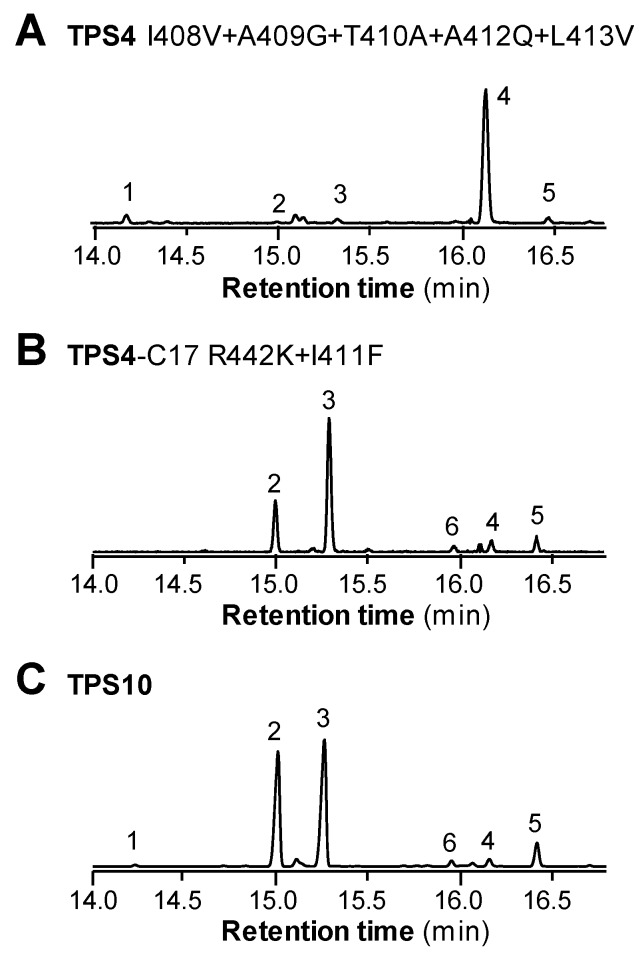
Active site amino acids and residues adjacent to them determine the different product specificities of TPS4 and TPS10. Product profiles of the mutants TPS4 I408V + A409G + T410A + A412Q + L413V (**A**) and TPS4-C17 R442K + I411F (**B**) and wild-type TPS10 (**C**) are shown. Genes were heterologously expressed in *Escherichia coli* and partially purified enzymes were incubated with (*E,E*)-farnesyl diphosphate (FPP) in the presence of the cofactor Mg^2+^. Total ion chromatograms of gas chromatography-mass spectrometry (GC-MS) analyses of the terpene products are shown. The sesquiterpene olefins were identified by mass spectrometry and comparison of mass spectra and retention times to those of authentic standards. 1, 7-*epi*-sesquithujene; 2, (*E*)-α-bergamotene; 3, (*E*)-β-farnesene; 4, β-bisabolene; 5, β-sesquiphellandrene; 6, zingiberene.

TPS4 and the closely related maize terpene synthase TPS5 form the same complex mixture of sesquiterpenes, but with different proportions of products [4]. These differences are due to four amino acid substitutions that likely determine the stereochemistry of the C6-C1 ring closure from the farnesyl cation to the bisabolyl cation. Models of the active site of TPS4 showed that the four amino acids are located in helix G1 and helix G2, which form the bottom of the active site cavity [10]. Notably, six of the active site amino acids that differ between TPS4 and TPS10 are positioned in the G1/G2 helices as well (Figure 2). In the course of the stepwise mutagenesis already described, three of these residues (A409G, T410A, L413V) were mutated in the background of an inactive combined mutant (TPS4-c14) leading to the active enzyme TPS4-c17, which exhibited product specificity similar to that of TPS10 (Figure 3B). In order to test whether the different product specificities of TPS4 and TPS4-c17 are determined just by the mutations in helices G1/G2, we introduced the respective amino acid changes into the wild-type TPS4 (Supplemental Table S1). However, the resulting enzyme TPS4-G2 formed mainly β-bisabolene and only trace amounts of (*E*)-β-farnesene and (*E*)-β-bergamotene (Figure 4A), indicating that amino acid changes in other regions of the active site also influence product specificity.

### 2.4. Residues Adjacent to Active Site Amino Acids Can Fine-Tune Product Specificity and Alter Turnover Number

**Figure 5:**
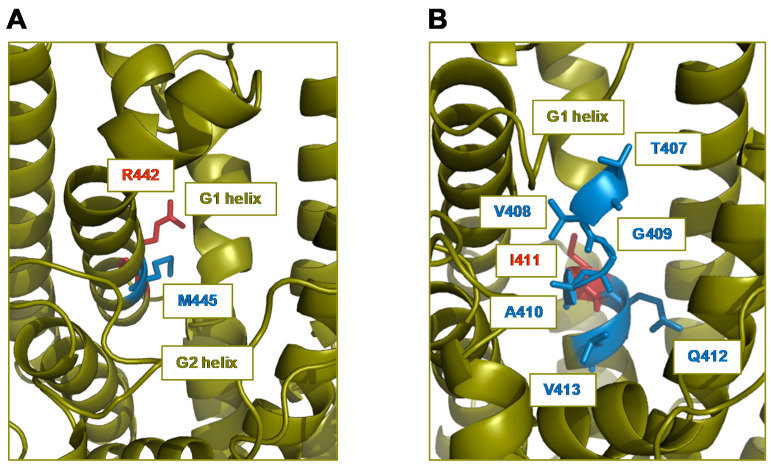
Model of the three-dimensional structure of the mutant TPS4-c17. The homology-based model was created using the crystal structure of 5-*epi*-aristolochene synthase from tobacco as template and allowed the identification of arginine 442 (**A**) and isoleucine 411 (**B**) as non-active site residues potentially influencing the product specificity. The pictures illustrate different views into the active site cavity. Residues lining the cavity are shown in blue and adjacent residues are shown in red. The helices G1 and G2 are labeled.

Although the mutant TPS4-c17 showed a product profile very similar to that of TPS10, the two enzymes exhibited differences in the ratio of products and catalytic efficiency (Figure 3B, Table 1 and Table 2). Since all the active site residues of the two enzymes are identical, amino acids outside the active site cavity must also affect catalysis. Because residues in the helices G1 and G2 had previously been shown to greatly influence the product outcome of TPS4 [4,10] (Figure 4A), we searched for amino acids outside the active site cavity that may directly interact with these helices. By visual inspection of a structure model of TPS4-c17, an arginine at position 442 was identified whose side chain was found to be oriented towards the G1 helix and thus may influence its position and orientation (Figure 5A). Moreover, arginine 442 is adjacent to methionine 445, one of the active site amino acids that was introduced instead of a glycine when TPS4-c17 was created from wild-type TPS4 (Figure 4A). In order to investigate the role of the residue at position 442, we created the mutant TPS4-c17 R442K by replacing arginine 442 with lysine, a residue located at the corresponding position in TPS10 (Figure 2). Heterologous expression and characterization of the mutant protein showed that the R442K replacement only marginally shifted the product specificity towards that of TPS10 (Table 1).

Another amino acid that potentially influences the conformations of the helices G1 and G2 is isoleucine 411. This residue is located at the N-terminal end of helix G2, but its side chain is directed opposite to the active site cavity and not directly involved in forming their contour (Figure 5B). Instead of the relatively small isoleucine in TPS4, TPS10 possesses a phenylalanine at this position that may affect the overall orientation of helix G2 and thus the conformation of active site amino acids. Although the mutation of isoleucine 411 to phenylalanine in TPS4-c17 shifted its product specificity towards enhanced formation of (*E*)-α-bergamotene, the quantitative composition of the resulting product profile was not identical compared to TPS10 (Figure 4B, Table 1). Interestingly, the double mutant containing the I411F and R442K replacements in the TPS4-c17 background showed an additive effect with an (*E*)-α-bergamotene/(*E*)-β-farnesene ratio near to that of TPS10 (Table 1). The *k*_cat_ value of TPS4-c17 R442K I411F was four times higher in comparison to TPS4-c17, but still ten times lower in comparison to TPS10 (Table 2; Supplemental Figure S2).

## 3. Discussion

The maize terpene synthases TPS4 and TPS10 form sesquiterpene mixtures that dominate the volatile blends of husk leaves and herbivore-damaged seedlings, respectively [4,5,6]. Although sharing only 57% amino acid sequence similarity, TPS4 and TPS10 produce structurally related compounds. The initial steps of their complex reaction sequences are identical and include the ionization of the substrate (*E,E*)-FPP, the isomerization of the formed farnesyl cation and the C6-C1 ring closure leading to the bisabolyl cation (Figure 1). Except for (*E*)-β-farnesene, which is formed by deprotonation of the initially generated farnesyl cation, all other TPS4 and TPS10 sesquiterpenes are derived from the bisabolyl cation by different hydride shifts and cyclizations. In order to understand the structural basis underlying the different product specificities of TPS4 and TPS10, we aimed to convert TPS4 into an enzyme having TPS10 activity by changing all 17 active site amino acids that differ between the two proteins. The mutant enzyme generated, TPS4-c17, indeed produced mainly the TPS10 products (*E*)-β-farnesene and (*E*)-α-bergamotene and only trace amounts of the TPS4 products 7-*epi*-sesquithujene and β-bisabolene. However, the (*E*)-β-farnesene/(*E*)-α-bergamotene ratio and the *k*_cat_ value were different in comparison to those for TPS10 (Table 1 and Table 2; Supplemental Figure S2). These results demonstrate that the product specificity as well as the catalytic efficiency of TPS4 and TPS10 are not exclusively determined by active site amino acids. In a study similar to ours, Greenhagen and colleagues compared two closely related sesquiterpene synthases, 5-*epi*-aristolochene synthase (TEAS) from *Nicotiana tabacum* and premnaspirodiene synthase (HPS) from *Hyoscyamus muticus* and identified the nine amino acids most important in determining product specificity [12]. Among the nine residues were amino acids that line the active site, but also residues without any contact to the active site cavity. Since the product specificities of TEAS and HPS could only be interconverted by changing all nine amino acids, the authors argue that amino acids adjacent to the active site help shape the active site geometry and modulate its dynamics [12]. We could also identify two amino acids outside the active sites of TPS4 and TPS10 that influence the catalytic outcome of the enzymes (Table 1 and Table 2). Although a single exchange of each of these residues had little effect on the product specificity, a combination of both mutations in the background of TPS4-c17 led to an enzyme with activity highly similar to that of TPS10. Similar additive effects of successive mutations of non-active site residues have also been reported for the TEAS-HPS system [12,13].

It has been shown that amino acids located in the G1-G2 helices, which form the bottom of the active site cavity, can influence the product specificity of terpene synthases [4,14,15,16,17,18,19]. Maize TPS4 and the closely related TPS5, for example, were completely interconvertible by switching four amino acids in this region [4]. Chiral analysis of reaction products and in silico substrate docking suggest that the four residues determine the stereospecificity of the C6-C1 ring closure [4,10]. Mutation of residues in the G1-G2 helices of other terpene synthases has also been reported to influence the initial cyclization of the farnesyl cation. For example, a mutation of glycine 402 in the germacrene A synthase (C10-C1 closure) from *Solidago canadensis* led to an enzyme that produced mainly γ-humulene (C11-C1 closure) [19]. The same effect, formation of γ-humulene, could be observed when threonine 399 in the α-bisabolol synthase (C6-C1 closure) of *Artemisia annua* was replaced by a leucine [18]. However, single exchanges of the G1-G2 helix residues A409, T410, A412 and L413 as well as the combined mutation of these residues in TPS4 mainly reduced the formation of the bicyclic 7-*epi*-sesquithujene, while the monocyclic β-bisabolene was still produced (Figure 3A and Figure 4A). This indicates that the different residues in the G1-G2 helices of TPS4 and TPS10 mainly mediate later steps of the reaction sequence such as hydride shifts and subsequent ring closures.

TPS crystal structures and molecular dynamics simulations demonstrated that a flexible loop between the helices J and K is important for catalytic activity [11,20]. After substrate binding, the J-K loop changes its conformation and closes the active site cavity. In the closed form, amino acids of the J-K loop can interact with the substrate and possibly participate in Mg^2+^ coordination [21,22]. Since the release of the product from the active site into the surrounding medium seems to be the rate-limiting step in the overall TPS reaction [23], dynamic changes of the J-K loop conformation necessary for opening the active site cavity may influence the reaction speed of terpene synthases. The mutant TPS4-c17 R442K + I411F showed a product profile highly similar to that of TPS10 (Table 1), however, its *k*_cat_ value was 10 times lower than that of TPS10 (Table 2; Supplemental Figure S2). The substantial amino acid differences in the J-K loop regions of TPS4 and TPS10 (Figure 1) could explain the observed kinetic effects. Changing the complete J-K loop in TPS4-c17 is thus a worthwhile aim for future studies to learn more about the role of this structure in terpene synthase catalysis.

## 4. Materials and Methods

### 4.1. cDNA Clones

The cDNA clones of TPS4 [4] and TPS10 [6] were characterized previously. Their sequences are deposited in GenBank with the accession numbers AY518310 (*tps4*-B73) and AY928078 (*tps10*-B73).

### 4.2. Modeling

Protein structure modeling was performed with the SWISS-MODEL service [24] using the previously determined structure of 5-*epi*-aristolochene synthase TEAS (5EAT) [11] as a modeling template. Models were visualized and analyzed using the Swiss-PdbViewer version 3.7 (Available online: https://spdbv.vital-it.ch/ (accessed on 24 April 2020)) and the software UCSF Chimera (Available online: https://www.cgl.ucsf.edu/chimera/ (accessed on 24 April 2020)).

### 4.3. Site-Directed Mutagenesis

For site-directed mutagenesis, the QuickChange site-directed mutagenesis kit (Stratagene, La Jolla, CA, USA) was used according to the manufacturer’s instruction. To create the single mutations in tps4, the PCR-based mutagenesis protocol was performed with the *tps4*-B73 cDNA cloned into the expression vector pASK-IBA7 [4] using primers containing the desired mutations (Supplemental Table S2). The combined mutants (Supplemental Table S1) were generated by an iterative approach using the preceding mutant as template for the next mutagenesis PCR (for primer information see Supplemental Table S2). To avoid a potential accumulation of random PCR errors in the vector sequence of the combined mutants, the ORF was amplified from the expression construct after 12 rounds of mutations and again cloned as a *Bsp*MI fragment into a freshly prepared pASK-IBA7 vector. The mutagenized constructs were fully sequenced before expression.

### 4.4. Subcloning of Tps Genes into the Expression Vector pHIS8-3

In order to obtain highly purified recombinant proteins for kinetic characterization, the ORFs of TPS4, TPS4-c17 and TPS4-c17 R442K I411F were amplified with the primers 5′-CCAGAATTCTCTTCGACCTTTCACCCAAGTCTG-3′ (forward) and 5′-ATAGTTTAGCGGCCGCTCATTCGGGTATTGGCTCCACAAAC-3′ (reverse) and cloned as *Eco*RI/*Not*I fragments into the expression vector pHIS8-3 [25]. The ORF of TPS10 was amplified with the primers 5′-TGCCATGGCGATGCCACCGCCTTCCAC-3′ (forward) and 5′-GGAATTCCCTAGAATAATGATATTGGATCCAC-3′ (reverse) and inserted as an *Nco*I/*Eco*RI fragment into the vector pHIS8-3.

### 4.5. Protein Overexpression and Enzyme Assay

Expression of pASK-IBA7 constructs and partial purification of recombinant proteins followed the procedure described in [4]. The pHIS8-3 constructs were transformed into *Escherichia coli* BL21 (λDE3) and liquid cultures were grown at 37 °C to an OD600 of 0.6. For induction of expression, 1-mM isopropyl β-D-thiogalactopyranoside (IPTG) was added. After 20 h incubation at 18 °C, the cells were harvested by centrifugation, resuspended in chilled lysis buffer (50-mM Tris-HCl (pH 8.0), 500-mM NaCl, 20-mM imidazole (pH 8.0), 10-mM β-mercaptoethanol, 1% (*v*/*v*) Tween 20, 10% (*v*/*v*) glycerol) and stirred for 2 h at 4 °C with lysozyme (0.5 mg/mL). After sonication and centrifugation, the supernatant was passed over a Ni^2+^-NTA-column (Qiagen, Hilden, Germany) and washed with 10 bed volumes of chilled lysis buffer and 10 bed volumes of chilled wash buffer (50-mM Tris-HCl (pH 8.0), 500-mM NaCl, 20-mM imidazole (pH 8.0), 10-mM β-mercaptoethanol, 10% (*v*/*v*) glycerol). The recombinant His tagged protein was eluted with 3 bed volumes of chilled elution buffer (50-mM Tris-HCl (pH 8.0), 500-mM NaCl, 250-mM imidazole (pH 8.0), 10-mM β-mercaptoethanol, 10% (*v*/*v*) glycerol) and transferred into reaction buffer (50-mM Tris-HCl (pH 8.0), 250-mM NaCl, 10-mM β-mercaptoethanol) using a NAPTM 5 column (Amersham Biosciences, Uppsala, Sweden). The protein concentration was determined by the method of Bradford using the Bio-Rad reagent (Bio-Rad, Hercules, CA, USA) with BSA as standard.

To analyze the catalytic activity of terpene synthase mutants in the vector pASK-IBA7, enzyme assays containing 50 µL of the bacterial extract and 50 µL assay buffer (10-mM Mopso (pH 7.0), 1-mM dithiothreitol, 10% (*v*/*v*) glycerol) with 10-µM (*E,E*)-FPP (Echelon Biosciences, Salt Lake City, UT, USA), 10-mM MgCl_2_, 0.2-mM NaWO_4_ and 0.1-mM NaF in a Teflon-sealed, screw-capped 1 mL GC glass vial were performed. A solid phase microextraction (SPME) fiber consisting of 100-µM polydimethylsiloxane (Supelco, Bellefonte, PA, USA) was placed into the headspace of the vial for 30 min incubation at 30 °C. For analysis of the adsorbed reaction products, the SPME fiber was directly inserted into the injector of the gas chromatograph.

For the kinetic characterization, assays containing purified recombinant His tagged protein, 10 μM [1-^3^H] (*E,E*)-farnesyl diphosphate (37 GBq mol-1, American Radiolabeled Chemicals, St. Louis, MO, USA) and 10-mM MgCl_2_ in 100 µL assay buffer were used. The assays were overlaid with 1 mL pentane to trap volatile products and incubated for 10 min at 30 °C. The reaction was stopped by mixing, and 0.5 mL of the pentane layer was taken for measurement of radioactivity by liquid scintillation counting in 2 mL Lipoluma cocktail (Packard Bioscience, Groningen, The Netherlands) using a Packard Tricarb 2300TR liquid scintillation counter (^3^H efficiency = 61%). The Km values were determined using seven substrate concentrations with four repetitions each. In all kinetic studies, enzyme concentration and incubation times were chosen so that the reaction velocity was linear during the incubation time period.

### 4.6. Gas Chromatography

A Hewlett-Packard model 6890 gas chromatograph was employed with the carrier gas He at 1 mL min^−1^, splitless injection (injector temperature 220 °C, injection volume 1 µL), a Chrompack CP-SIL-5 CB-MS column ((5%-phenyl)-methylpolysiloxane, 25 m × 0.25 mm i.d. × 0.25-μm film thickness, Varian, Palo Alto, CA, USA) and a temperature program from 40 °C (3-min hold) at 5 °C min^−1^ to 240 °C (3 min hold). The coupled mass spectrometer was a Hewlett-Packard model 5973 with a quadrupole mass selective detector, transfer line temperature 230 °C, source temperature 230 °C, quadrupole temperature 150 °C, ionization potential 70 eV, and a scan range of 40–350 atomic mass units. For the accurate measurement of TPS product composition, samples were analyzed by gas chromatography with a flame ionization detector (FID) operated at 250 °C using conditions as above, except that carrier gas was H_2_ at 2 mL min^−1^. Compounds produced by TPS4, TPS10, and the mutant enzymes were identified as described [4,6].

### 4.7. Antibodies, Gel Electrophoresis and Immunoblotting

Polyclonal antibodies for TPS4 were generated in rabbits using two synthetic peptides corresponding to residues 58 to 72 and 455 to 469 (Eurogentec, Herstal, Belgium). In order to determine the amount of soluble recombinant protein in the bacterial extracts, 10 µL of crude extract were resolved by SDS PAGE and transferred onto a Hybond-XL nylon membrane (Amersham Biosciences) using a Bio-Rad blotting apparatus with blot buffer (25-mM Tris-HCl, 192-mM glycine, 20% (*v*/*v*) methanol). The membrane was blocked with 2% (*w/v*) bovine serum albumin in TBST (10-mM Tris-HCl (pH 8.0), 150-mM NaCl, 0.05% (*v*/*w*) Tween 20) and then incubated first with the polyclonal antiserum (1:500 in TBST) and then with alkaline phosphatase-conjugated antirabbit IgG (Sigma, Deisenhofen, Germany). For visualization, the NBT/BCIP Liquid Substrate system (Sigma) was used.

### 4.8. Statistical Analysis

Statistical analysis was performed with SigmaPlot 11.0 for Windows (Systat Software Inc., San Jose, California). The data were log transformed to meet statistical assumptions such as normality and homogeneity of variances. Differences between *k*_cat_ values were analyzed by a one-way ANOVA and a post hoc test (Tukey’s test). All relevant parameters of the statistical analysis including *p* values are shown in Supplemental Figure S2.

## Figures and Tables

**Table 1:** Composition of sesquiterpene mixtures formed by TPS4-c17, TPS4-c17 R442K, TPS4-c17 I411F, TPS4-c17 R442K + I411F and TPS10. Mean values and standard errors (*n* = 4 technical replicates) are shown.

Product Name	TPS4-c17 (%)	TPS4-c17 R442K (%)	TPS4-c17 I411F (%)	TPS4-c17 R442K + I411F (%)	TPS10 (%)
7-*epi*-sesquithujene	0.18 ± 0.06	0.35 ± 0.09	0.56 ± 0.07	0.28 ± 0.05	0.51 ± 0.03
(*E*)-α-bergamotene	14.41 ± 0.20	16.06 ± 1.15	18.78 ± 0.19	20.97 ± 1.02	35.85 ± 0.30
sesquisabinene A	1.85 ± 0.13	1.10 ± 0.16	1.39 ± 0.10	1.21 ± 0.08	2.08 ± 0.03
(*E*)-β-farnesene	61.54 ± 0.42	59.67 ± 0.55	56.16 ± 0.15	56.52 ± 1.28	50.41 ± 0.34
zingiberene	4.37 ± 0.25	3.68 ± 0.48	4.23 ± 0.06	3.92 ± 0.06	1.61 ± 0.04
β-bisabolene	9.91± 0.06	9.99 ± 0.71	7.48 ± 0.23	7.44 ± 0.26	2.24 ± 0.10
β-sesquiphellandrene	7.74 ± 0.14	9.15 ± 0.38	11.40 ± 0.44	9.67 ± 0.13	7.31 ± 0.14

**Table 2:** Kinetic constants of TPS4, TPS4-c17, TPS4-c17 R442K + I411F and TPS10. Mean values and standard errors are shown (*n* = 4 technical replicates).

Kinetic Parameter	TPS4	TPS4-c17	TPS4-c17 R442K + I411F	TPS10
***K*_m_** (µM)	3.8 ± 0.5	2.1 ± 0.3	2.3 ± 0.3	3.2 ± 0.5
***k*_cat_** (s^−1^)	(1.05 ± 0.06) × 10^−2^	(4.55 ± 0.27) × 10^−3^	(2.42 ± 0.02) × 10^−2^	(2.04 ± 0.13) × 10^−1^

## Supplementary Materials

The following are available online at https://www.mdpi.com/2223-7747/9/5/552/s1, Figure S1: Three-dimensional model of the maize terpene synthase TPS4. The homology-based model was created using the crystal structure of the 5-*epi*-aristolochene synthase from tobacco [13] as template. Amino acids lining the active site cavity are shown in orange. The N-terminal and C-terminal domains are highlighted in blue and green, respectively, Figure S2: Statistical parameters of the one-way ANOVA analysis of *k*_cat_ values, Table S1: Mutant proteins generated and analyzed in this study, Table S2: Primer sequences for site-directed mutagenesis.
