# Supplementary material for: The Product Specificities of Maize Terpene Synthases TPS4 and TPS10 Are Determined Both by Active Site Amino Acids and Residues Adjacent to the Active Site
Source: Plants (Basel). 2020 Apr 26;9(5):552. doi: 10.3390/plants9050552 (PMC7284416; doi:10.3390/plants9050552)
Supplement: Supplementary file 1 [file plants-09-00552-s001.zip › 2020-04-21 supplemental tables.docx]

**Supplemental Tables**

**Supplemental Table 1.** Mutant proteins generated and analyzed in this study.

| **Name** | **Introduced mutation** (in TPS4) |
| --- | --- |
| TPS4-s1  TPS4-s3  TPS4-s4  TPS4-s5  TPS4-s6  TPS4-s7  TPS4-s8  TPS4-s9  TPS4-s10  TPS4-s11  TPS4-s12  TPS4-s13  TPS4-s14  TPS4-s16  TPS4-s17  TPS4-G2  TPS4-c3  TPS4-c4  TPS4-c5  TPS4-c6  TPS4-c7  TPS4-c8  TPS4-c9  TPS4-c10  TPS4-c11  TPS4-c12  TPS4-c13  TPS4-c14  TPS4-c16  TPS4-c17  TPS-c17 I411F  TPS-c17 R442K  TPS-c17 R442K+I411F | Y382S  A533G, L534F  T524A  P467V  K463A  V455A  V448S  G445M  A412Q  I408V  A405G  G302A  E277C  A409G, T410A  L413V  I408V, A409G, T410A, A412Q, L413V  Y382S, A533G, L534F  Y382S, A533G, L534F, T524A  Y382S, A533G, L534F, T524A, P467V  Y382S, A533G, L534F, T524A, P467V, K463A  Y382S, A533G, L534F, T524A, P467V, K463A, V455A  Y382S, A533G, L534F, T524A, P467V, K463A, V455A, V448S  Y382S, A533G, L534F, T524A, P467V, K463A, V455A, V448S, G445M  Y382S, A533G, L534F, T524A, P467V, K463A, V455A, V448S, G445M, A412Q  Y382S, A533G, L534F, T524A, P467V, K463A, V455A, V448S, G445M, A412Q, I408V  Y382S, A533G, L534F, T524A, P467V, K463A, V455A, V448S, G445M, A412Q, I408V, A405G  Y382S, A533G, L534F, T524A, P467V, K463A, V455A, V448S, G445M, A412Q, I408V, A405G, G302A  Y382S, A533G, L534F, T524A, P467V, K463A, V455A, V448S, G445M, A412Q, I408V, A405G, G302A, E277C  Y382S, A533G, L534F, T524A, P467V, K463A, V455A, V448S, G445M, A412Q, I408V, A405G, G302A, E277C, A409G, T410A  Y382S, A533G, L534F, T524A, P467V, K463A, V455A, V448S, G445M, A412Q, I408V, A405G, G302A, E277C, A409G, T410A, L413V  Y382S, A533G, L534F, T524A, P467V, K463A, V455A, V448S, G445M, A412Q, I408V, A405G, G302A, E277C, A409G, T410A, L413V, I411F  Y382S, A533G, L534F, T524A, P467V, K463A, V455A, V448S, G445M, A412Q, I408V, A405G, G302A, E277C, A409G, T410A, L413V, R442K  Y382S, A533G, L534F, T524A, P467V, K463A, V455A, V448S, G445M, A412Q, I408V, A405G, G302A, E277C, A409G, T410A, L413V, I411F, R442K |

**Supplemental Table 2.** Primer sequences for site-directed mutagenesis.

| **Mutation^a^** | **Sequence (5'/3')^b^** | **Direction^c^** |
| --- | --- | --- |
| Y382S | CGTTTAGTTGAGCTAT**C**CTCCAAGGAAATAAA | fwd |
| Y382S | TTTATTTCCTTGGAG**G**ATAGCTCAACTAAACG | rev |
| A533G+L534V | CAGGGATCGTGACG**G**ATT**C**ACCTCTTCGGAAGC | fwd |
| A533G+L534V | GCTTCCGAAGAGGT**G**AAT**C**CGTCACGATCCCTG | rev |
| T524A | GACTTCTCAAGAACC**G**CGGATAATATGTACAG | fwd |
| T524A | CTGTACATATTATCCG**C**GGTTCTTGAGAAGTC | rev |
| P467V | CAAACAAAAGACCATAGT**GT**CTCCACTGTCCATTG | fwd |
| P467V | CAATGGACAGTGGAG**AC**ACTATGGTCTTTTGTTTG | rev |
| K463A^s^ | CAAGCGTGAGCAAACA**GC**AGACCATAGTCCCTC | fwd |
| K463A^s^ | GAGGGACTATGGTCT**GC**TGTTTGCTCACGCTTG | rev |
| K463A^m^ | CAAGCGTGAGCAAACA**GC**AGACCATAGT**GT**CTCC | fwd |
| K463A^m^ | GGAG**AC**ACTATGGTCT**GC**TGTTTGCTCACGCTTG | rev |
| V455A | CTCTCCAACGATGTCG**C**ATCGACCAAGCGTGAG | fwd |
| V455A | CTCACGCTTGGTCGAT**G**CGACATCGTTGGAGAG | rev |
| V448S | ACTTTTGGTTCATTT**TC**ACGGCTCTCCAACGATG | fwd |
| V448S | CATCGTTGGAGAGCCGT**GA**AAATGAACCAAAAGT | rev |
| G445M^s^ | TTTATAAGAACTTTT**ATG**TCATTTGTACGGCTC | fwd |
| G445M^s^ | GAGCCGTACAAATGA**CAT**AAAAGTTCTTATAAA | rev |
| G445M^m^ | TTTATAAGAACTTTT**ATG**TCATTT**TC**ACGGCTC | fwd |
| G445M^m^ | GAGCCGT**GA**AAATGA**CAT**AAAAGTTCTTATAAA | rev |
| A412Q | GACCATCGCAACCATT**CAG**CTAACATGTTCTGCAT | fwd |
| A412Q | ATGCAGAACATGTTAG**CTG**AATGGTTGCGATGGTC | rev |
| I408V^s^ | GTTTCAGCAGAGACC**G**TCGCAACCATTGCTC | fwd |
| I408V^s^ | GAGCAATGGTTGCGA**C**GGTCTCTGCTGAAAC | rev |
| I408V^m^ | GTTTCAGCAGAGACC**G**TCGCAACCATT**CAG**C | fwd |
| I408V^m^ | G**CTG**AATGGTTGCGA**C**GGTCTCTGCTGAAAC | rev |
| A405G^s^ | CCTCCAGGTTTCAG**G**AGAGACCATCGCAAC | fwd |
| A405G^s^ | GTTGCGATGGTCTCT**C**CTGAAACCTGGAGG | rev |
| A405G^m^ | CCTCCAGGTTTCAG**G**AGAGACC**G**TCGCAACC | fwd |
| A405G^m^ | GGTTGCGA**C**GGTCTCT**C**CTGAAACCTGGAGG | rev |
| G302A | TGACAAAGATCACGG**C**ACTTATTACAATAATC | fwd |
| G302A | GATTATTGTAATAAGT**G**CCGTGATCTTTGTCA | rev |
| E277C | GAGATAGAATTGTAGAG**TGT**TATTTCTGGATGAATG | fwd |
| E277C | CATTCATCCAGAAATA**ACA**CTCTACAATTCTATCTC | rev |
| A409G+T410A^s^ | CAGCAGAGACCATCG**G**A**G**CCATTGCTCTAACATG | fwd |
| A409G+T410A^s^ | CATGTTAGAGCAATGG**C**T**C**CGATGGTCTCTGCTG | rev |
| A409G+T410A^m^ | CAG**G**AGAGACC**G**TCG**G**A**G**CCATT**CAG**CTAACATG | fwd |
| A409G+T410A^m^ | CATGTTAG**CTG**AATGG**C**T**C**CGA**C**GGTCTCT**C**CTG | rev |
| L413V^s^ | ATCGCAACCATTGCT**G**TAACATGTTCTGCAT | fwd |
| L413V^s^ | ATGCAGAACATGTTA**C**AGCAATGGTTGCGAT | rev |
| L413V^m^ | **G**TCG**G**A**G**CCATT**CAGG**TAACATGTTCTGCATA | fwd |
| L413V^m^  R442K^m^  R442K^m^  I411F^m^  I411F^m^  R442K+I411F^m^  R442K+I411F^m^ | TATGCAGAACATGTTA**CCTG**AATGG**C**T**C**CGA**C**  CTTTCCTCAATTTATAA**A**AACTTTTATGTCATTTTC  GAAAATGACATAAAAGTT**T**TTATAAATTGAGGAAAG  GAGACC**G**TCG**G**A**G**CC**T**TT**CAGG**TAACATGTTC  GAACATGTTAcCTGAA**A**GGCTCCGACGGTCTC  CTTTCCTCAATTTATAA**A**AACTTTTATGTCATTTTC  GAAAATGACATAAAAGTT**T**TTATAAATTGAGGAAAG | rev  fwd  rev  fwd  rev  fwd  rev |

^a^ primers marked with ^s^ were used to create the single mutations TPS4-s1 – TPS4-s17 whereas primers marked with ^m^ were used to create the combinatorial mutants TPS4-c1 – TPS4-c19

^b^ The base changes are shown in boldface and underlined

^c^ fwd, forward; rev, reverse
