# Supplementary material for: The Product Specificities of Maize Terpene Synthases TPS4 and TPS10 Are Determined Both by Active Site Amino Acids and Residues Adjacent to the Active Site
Source: Plants (Basel). 2020 Apr 26;9(5):552. doi: 10.3390/plants9050552 (PMC7284416; doi:10.3390/plants9050552)
Supplement: Supplementary file 1 [file plants-09-00552-s001.zip › 2020-04-22 supplemental figures.pptx]

## Slide 1
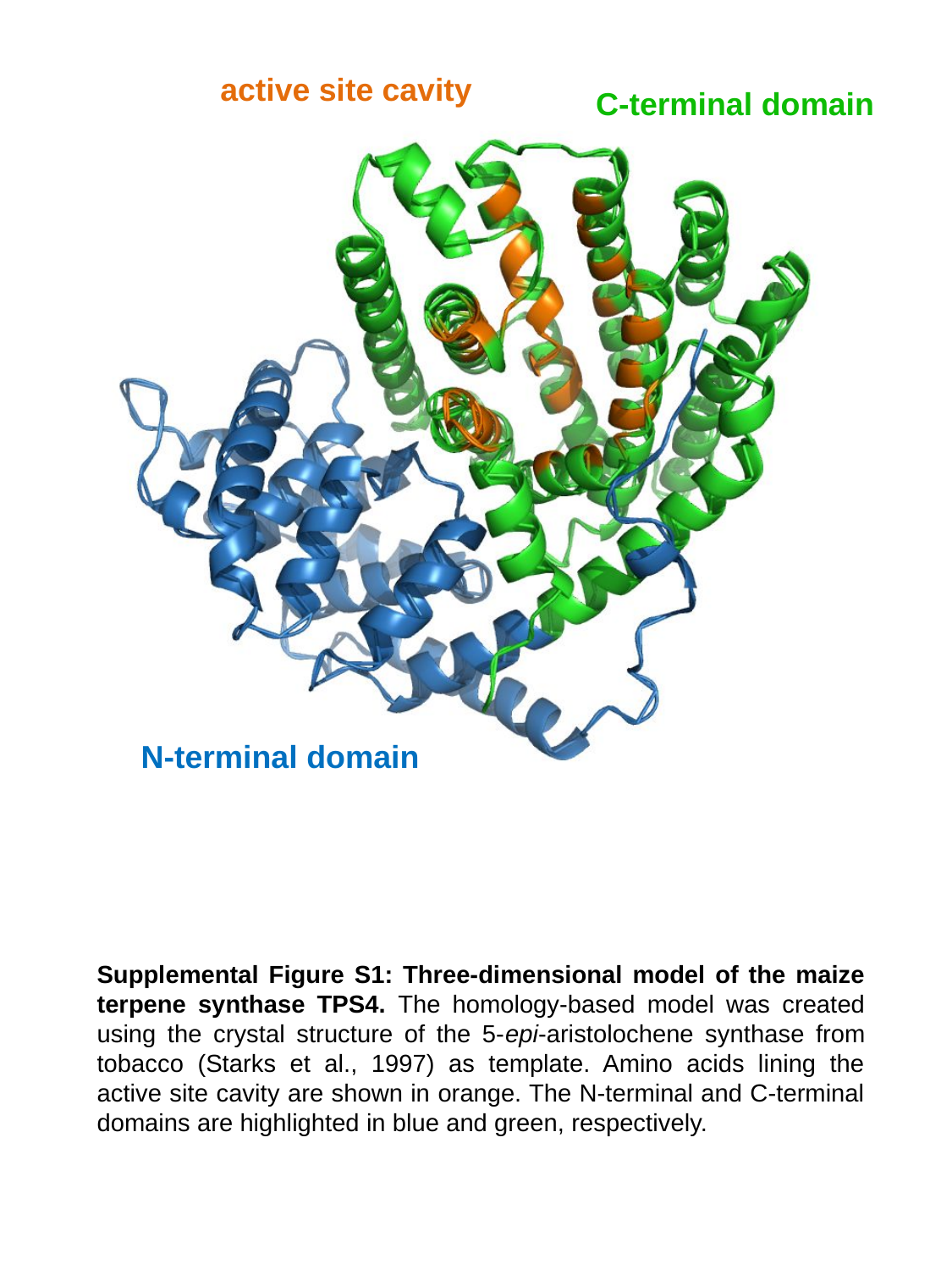

active site cavity
C-terminal domain
N-terminal domain
Supplemental Figure S1: Three-dimensional model of the maize terpene synthase TPS4. The homology-based model was created using the crystal structure of the 5-epi-aristolochene synthase from tobacco (Starks et al., 1997) as template. Amino acids lining the active site cavity are shown in orange. The N-terminal and C-terminal domains are highlighted in blue and green, respectively.

## Slide 2
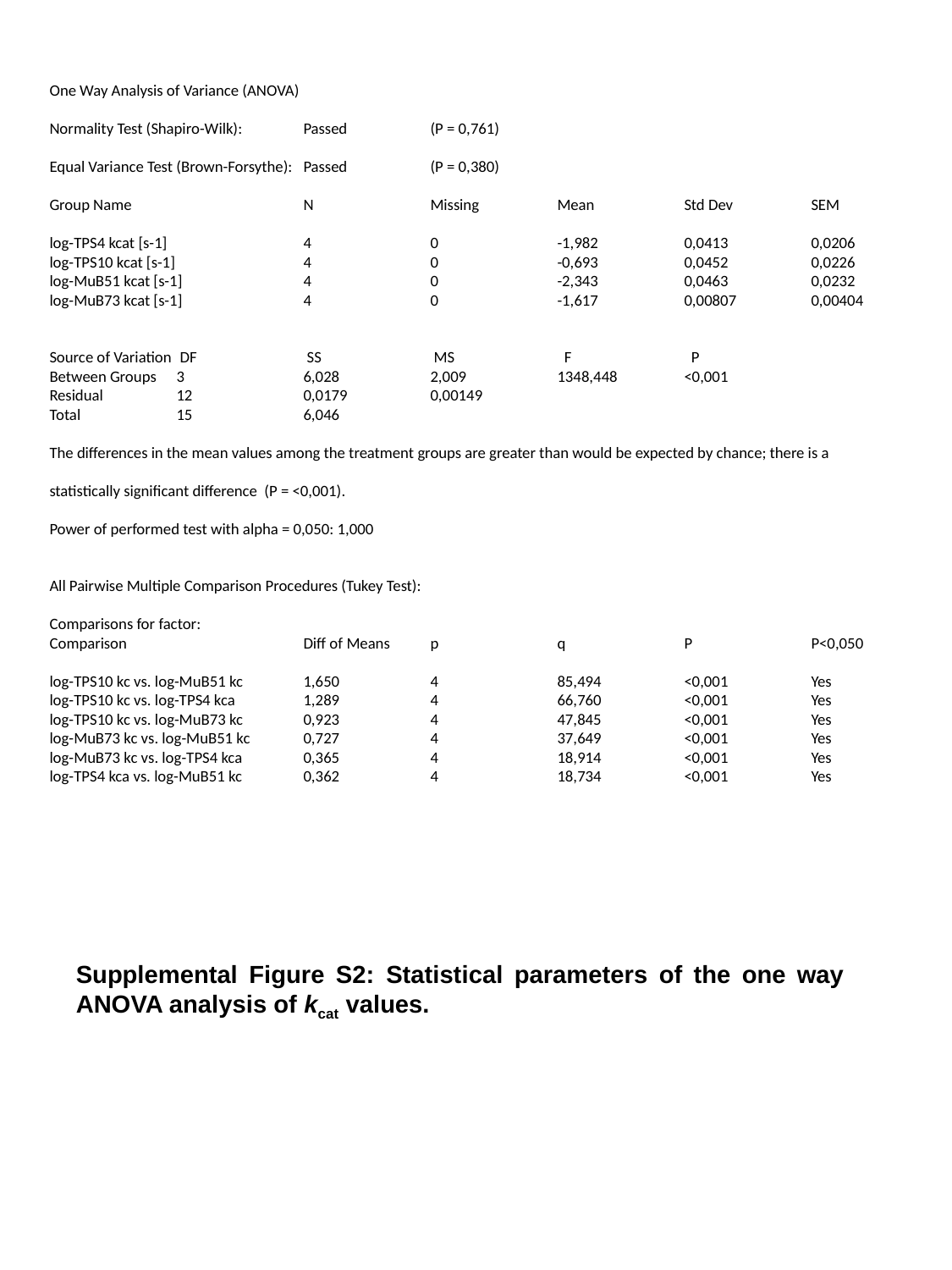

One Way Analysis of Variance (ANOVA)
Normality Test (Shapiro-Wilk): 	Passed	(P = 0,761)
Equal Variance Test (Brown-Forsythe):	Passed	(P = 0,380)
Group Name 		N 	Missing	Mean	Std Dev	SEM
log-TPS4 kcat [s-1]		4	0	-1,982	0,0413	0,0206
log-TPS10 kcat [s-1]		4	0	-0,693	0,0452	0,0226
log-MuB51 kcat [s-1]	4	0	-2,343	0,0463	0,0232
log-MuB73 kcat [s-1]	4	0	-1,617	0,00807	0,00404
Source of Variation	 DF 	 SS 	 MS 	 F 	 P
Between Groups	3	6,028	2,009	1348,448	<0,001
Residual	12	0,0179	0,00149
Total	15	6,046
The differences in the mean values among the treatment groups are greater than would be expected by chance; there is a
statistically significant difference (P = <0,001).
Power of performed test with alpha = 0,050: 1,000
All Pairwise Multiple Comparison Procedures (Tukey Test):
Comparisons for factor:
Comparison		Diff of Means	p	q	P	P<0,050
log-TPS10 kc vs. log-MuB51 kc	1,650	4	85,494	<0,001	Yes
log-TPS10 kc vs. log-TPS4 kca	1,289	4	66,760	<0,001	Yes
log-TPS10 kc vs. log-MuB73 kc	0,923	4	47,845	<0,001	Yes
log-MuB73 kc vs. log-MuB51 kc	0,727	4	37,649	<0,001	Yes
log-MuB73 kc vs. log-TPS4 kca	0,365	4	18,914	<0,001	Yes
log-TPS4 kca vs. log-MuB51 kc	0,362	4	18,734	<0,001	Yes
Supplemental Figure S2: Statistical parameters of the one way ANOVA analysis of kcat values.
